# Analysis of Waste Generation Characteristics during New Apartment Construction—Considering the Construction Phase

**DOI:** 10.3390/ijerph16183485

**Published:** 2019-09-19

**Authors:** Young-Chan Kim, Yuan-Long Zhang, Won-Jun Park, Gi-Wook Cha, Jung-Wan Kim, Won-Hwa Hong

**Affiliations:** 1 Innovative Durable Building and Infrastructure Research Center, Hanyang University, 55 Hanyangdaehak-ro, Sangnok-gu, Ansan 426-791, Korea; yyoungchani@gmail.com; 2 School of Architecture, Civil, Environmental and Energy Engineering, Kyungpook National University, 80 Daehak-ro, Buk-gu, Daegu 41566, Korea; showme123@hotmail.com; 3 Department of Civil Engineering, Kangwon National University, 346, Jungang-ro, Samcheok-si, Gangwon-do 25913, Korea; wjpark@kangwon.ac.kr; 4 Department of Architectural Engineering, Jeju National University, 102 Jejudaehak-ro, Jeju-si, Jeju Special Self-Governing Province 63243, Korea; cgwgnr@gmail.com; 5 POSCO E&C, Building Works Division, Building Technology Group 241, Incheon tower-daero, Yeonsu-gu, Incheon 22009, Korea; kjw7878@poscoenc.com

**Keywords:** waste generation rate (WGR), construction waste (CW), generation characteristics, new apartment construction

## Abstract

The waste generation rate (WGR) is used to predict the generation of construction and demolition waste (C&DW) and has become a prevalent tool for efficient waste management systems. Many studies have focused on deriving the WGR, but most focused on demolition waste rather than construction waste (CW). Moreover, previous studies have used theoretical databases and thus were limited in showing changes in the generated CW during the construction period of actual sites. In this study, CW data were collected for recently completed apartment building sites through direct measurement, and the WGR was calculated by CW type for the construction period. The CW generation characteristics by type were analyzed, and the results were compared with those of previous studies. In this study, CW was classified into six types: Waste concrete, waste asphalt concrete, waste wood, waste synthetic resin, waste board, and mixed waste. The amount of CW generated was lowest at the beginning of the construction period. It slowly increased over time and then decreased again at the end. In particular, waste concrete and mixed waste were generated throughout the construction period, while other CWs were generated in the middle of the construction period or towards the end. The research method and results of this study are significant in that the construction period was considered, which has been neglected in previous studies on the WGR. These findings are expected to contribute to the development of efficient CW management systems.

## 1. Introduction

The construction industry makes significant contributions to the national economic development through considerable job creation, inputs in the production process, and all sectors that produce equipment and services [1]. However, construction and demolition (C&D) activities generate not only construction and demolition waste (C&DW) but also a considerable environmental burden through increased environmental pollution, land quality degradation, and resource depletion [2,3]. Because C&DW is a very important issue at the national and municipal levels [1], its accurate estimation is very important for the development of efficient waste management systems [4,5]. Therefore, optimizing C&DW management (C&DWM) is recognized as a very important issue in the literature [6]. In particular, the waste generation rate (WGR) predicts the generation of C&DW and has become a prevalent tool for efficient waste management systems [7].

Many researchers have conducted studies on C&DW and WGR using various data collection and analysis methods. To derive the WGR, data must first be collected. Data collection methods can be classified into two types: Indirect (information is taken from input quantities for construction and previous studies, e.g., [8]) and direct (C&DW is measured onsite, e.g., [9]). Indirect measurement methods cannot reflect changes in material quantities due to building renovation, while direct measurement methods may risk data errors due to distortion of the C&DW information by contractors [10].

Next, the collected data must be evaluated and analyzed to derive the WGR. There are two representative methods for evaluating the WGR: by population and by building area. According to Yost and Halstead (1996) [11], C&DW is estimated according to the WGR per person in many cases. However, the WGR per person is significantly different for each country, which may cause actual spatial and visual differences for construction activities as well as differences in definitions and procedures during waste registration [12,13]. Owing to such drawbacks, the use of the WGR per person has been severely criticized in previous studies [11,14,15].

The quantification of C&DW generally depends on variables that reflect the size of the project, such as the financial value of building permits [11], quantities of input materials [16,17], and floor area of the constructed building [18,19,20,21]. Thus, the evaluation by building area is generally the most suitable for calculating the amount of C&DW generated [1]. A detailed analysis of the C&DW generation status can reduce the amount of generated waste and contribute to its efficient treatment and recycling. Therefore, research is needed on calculating the unit generation rates by classifying C&DW by use and structure [22]. In recently, there have been studies that estimate C&DW generation using Building Information Modeling (BIM; e.g., quantity take off), but they have limitations that cannot reflect various characteristics of actual construction sites (e.g., [23,24]).

The main purpose of this study was to analyze the construction waste (CW) generation characteristics during the construction period of an apartment building and compare the results with previous studies. In South Korea, 76% of the buildings constructed over the last decade are apartment buildings [25]. Most studies on the WGR of new construction sites were conducted between 1990 and the beginning of the 2010s. The time that has elapsed since them has created uncertainty over the data collection method, and the WGR needs to be reestablished. In addition, previous studies had limitations when reflecting changes in the generation of CW during a construction period. In this study, the WGR of each CW type was calculated from the direct measurement of trucks carrying out CW generated at construction sites, and the generation characteristics of each CW type during the construction period were analyzed. The analysis results were then compared with the results of previous studies. The methodology used in this study should be applicable to calculating the WGR for apartment buildings as well as other building types, and the results can be used as basic data for more systematic CW management.

## 2. Literature Review

### 2.1. Previous Studies on WGR

In much of the literature, the WGR was calculated to predict the amount of CW generated during construction. In general, WGR can be explained from two perspectives: (1) Classifying waste by type and (2) treating the entire waste amount as a whole [7]. While Method 1 enables the detailed investigation of certain types of waste depending on the building characteristics and treatment process/method by material, Method 2 can calculate the total amount of waste generated from one or several projects [26]. Therefore, the WGR can be calculated by properly selecting and applying the two methods depending on the scope of investigation. Cochran et al. (2007) [27] classified waste by type and calculated the WGR for each type with Method 1. Paz and Lafayette (2016) [1] used Method 2 and derived the WGR by calculating the amount of waste generated per square meter of the gross floor area (GFA) without classifying materials. In this study, the types of waste generated at the construction site of the target building were classified with Method 1, and the unit generation rate for each material was calculated.

The WGR can be expressed in various units considering the attributes of each material: (1) The percentage of each purchased material, (2) unit weight (kg/m^2^ of the GFA), and (3) unit volume (m^3^/m^2^ of the GFA) [26]. In the case of Unit 1, the amount of waste for each material can be calculated by multiplying the total amount of waste generated by the percentage of each material. Lachimpadi et al. (2012) [28] used Unit 1 and calculated the WGR by using the percentage of each material in the total waste. In the cases of Units 2 and 3, the weight (kg) or volume (m^3^) of the total waste can be calculated by multiplying the WGR (kg/m^2^ or m^3^/m^2^) by the GFA. Lage et al. (2010) [29] calculated the WGR (kg/m^2^) for each material with Unit 2. Solís-Guzmán et al. (2009) [17] calculated the WGR (m^3^/m^2^) for each material with Unit 3. The WGRs calculated in various units differ. When the waste generation at the regional level is predicted with Unit 1, the characteristics and types of various buildings in the region are not reflected. With Unit 3, errors may occur in the calculation results compared to Unit 2 because the shapes and volumes of the materials used differ depending on the characteristics or sizes of buildings. In this study, Unit 2 was used; thus, the WGR was evaluated according to the weight and GFA.

### 2.2. Data Collection Methods for WGR Calculation

The data collection methods for WGR calculation can be classified into two categories, as discussed in the introduction: Indirect measurement (IM) and direct measurement (DM). IM can be further classified into two categories as follows: (1) Summarizing the WGR databases (DBs) of the literature and previous studies and (2) calculating the input amounts of materials and addition rate of each material. DM determines the amount of waste generated through measurements by the contractor and direct measurements by the investigator. DM is disadvantageous compared to IM in terms of time and cost but can derive accurate WGR. In this study, an analysis was conducted after data were collected through DM by the investigator (the data were collected directly by the authors for measurement because data collection by a contractor might have resulted in the mixing of different waste types). Table 1 presents the advantages and disadvantages of the available data collection methods for WGR calculation.

### 2.3. Comparison of Data from Previous Studies

Related previous studies were collected and analyzed to calculate the WGR of CW generated by apartment buildings. The types of CW can be classified in various ways to calculate the WGR. In previous studies, C&DW generated at construction sites can be classified into 18 types: Waste concrete, waste asphalt concrete, waste brick, waste block, waste roofing tile, waste wood, waste synthetic resin, waste fiber, waste wallpaper, waste metal, waste glass, waste tile, waste board, waste panel, mixed waste, waste soil and stone, and construction sludge. There have been no recent studies on the WGR of CW, and recent building sizes or site characteristics have not been properly considered. Table 2 compares studies on the WGR of CW [24]. These previous studies exhibited significantly different WGRs and classified CW into 3–13 types. This is because the accuracy differed with the various data collection methods, and the waste classification differed depending on the laws or guidelines of each country or region.

## 3. Materials and Methods

**Figure 1:**
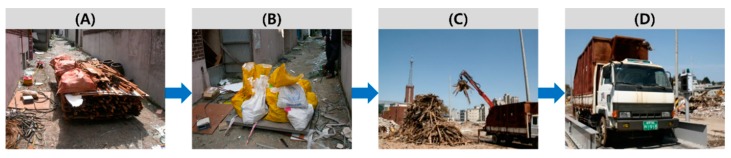
The data collection methods for WGR calculation: (**A**) Generated CW; (**B**) CW Type Classification; (**C**) Loading Dump Truck; (**D**) Weight Measurement.

In this study, CW DBs were collected from the data generated when a major construction company in South Korea built new apartment buildings. For data collection, first, the researchers of this study visited construction sites and classified CW by type. Next, the classified CW was loaded onto a dump truck using equipment. Finally, the weight of the truck loaded with the CW was measured and then the weight of the empty truck was subtracted to obtain the weight of the CW. Figure 1 shows the data collection methods for WGR calculation. Moreover, for the analysis of the collected DBs, the DBs comprised data collected on the amount and treatment status of CW generated at construction sites by day and by waste type during the construction period. The total construction period was divided into 10% segments, and the amounts of CW generated for each segment were compared to examine changes during the construction period. The collected DBs were from apartment buildings that were constructed after 2012. These were targeted for the following reasons: (1) Apartment buildings represent a high proportion of housing construction, and a future increase in CW generation is expected because of the new construction of apartment buildings; (2) most studies were conducted before 2012, so they cannot properly reflect the latest construction methods or building sizes and characteristics. Table 3 presents an overview of the target sites. The target sites were very large in size, so only three sites were investigated owing to limited time and manpower even though securing more DBs would be desirable.

In most previous studies, DBs for estimating the WGR were collected from government databases, building permits issued, statistical institutes, and handbooks [18,27,29,41]. However, the WGRs estimated from such DBs significantly differ from the actual amounts of CW generated onsite [26]. In this study, DM as described in Table 1 (measurement of the weights of trucks carrying out the CW generated at construction sites) was used to collect data for calculating the WGR. This is because DM can be used to collect data with significant accuracy and reliability, even though it requires much time and manpower. Based on the collected DBs, the WGR was calculated in terms of the unit weight (kg/m^2^ of the GFA), as presented in Section 2.1. In general, waste is carried out from construction sites using trucks and the cost is determined by the tonnage (e.g., 5-ton truck, 10-ton truck, and 20-ton truck) and number of trucks. Therefore, in this study, the results were compared by unit weight. WGR can be calculated using the following equation.

(1) $$WGR_{j}=\frac{\sum A_{ij}}{GFA}$$

where, *WGR_j_* is the unit generation rate of *j*-type CW; *A_ij_* is the amount of *j*-type CW generated from *i* site (kg); *GFA* is the gross for area (m^2^).

Although C&DW is generally classified into 18 types, as noted in Section 2.3, CW is discharged in mixed forms at actual sites, and onsite CW classification and discharge differ depending on the onsite characteristics [29]. In this study, CW was classified into eight types in consideration of the waste discharge characteristics of the target sites: Waste concrete, waste asphalt concrete, waste wood, waste synthetic resin, waste board, mixed waste, waste soil and stone, and construction sludge. Waste soil and stone and construction sludge were excluded from the main analysis because they are not necessarily generated at construction sites, unlike other CW types, and they may not be generated depending on the onsite characteristics. In other words, the analysis in this study focused on six CW types: Waste concrete, waste asphalt concrete, waste wood, waste synthetic resin, waste board, and mixed waste.

## 4. Results and Discussion

The characteristics of CW generated at new apartment construction sites (i.e., total amount of CW generated, and amount generated in each segment) during the construction period were analyzed with the DBs discussed in Section 3. The results were then compared with those of previous studies on the WGR of CW. The raw DBs additionally used in this study were attached to Supplementary Data (see Tables S1–S3).

### 4.1. General Analysis Results

**Figure 2:**
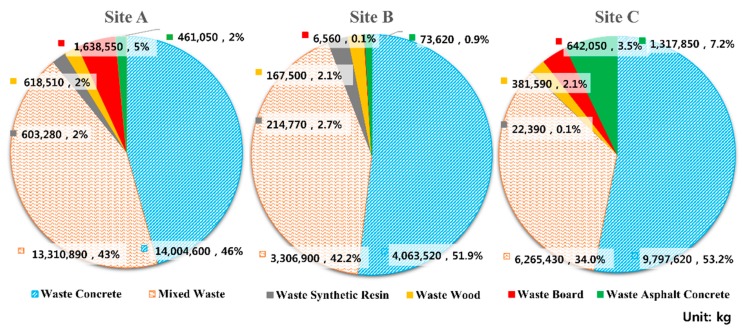
Generation amount by CW type at each site.

Table 4 presents the amount of each CW type generated at each site. In addition, Figure 2 shows the general analysis results in diagrams. The total amount of CW generated at the three sites was 56,896,680 kg (site A: 30,636,880 kg, site B: 7,832,870 kg, and site C: 18,426,930 kg). For site A, waste concrete made up the highest proportion (45.7%), followed by mixed concrete (43.4%), and waste board (5.3%). For Site B, waste concrete made up the highest proportion (51.9%), followed by mixed concrete (42.2%) and waste synthetic resin (2.7%). For Site C, waste concrete made up the highest proportion (53.2%), followed by mixed concrete (34.0%) and waste asphalt concrete (7.2%). In other words, waste concrete and mixed concrete represented the majority of CW at all of the sites. Table 5 presents the WGR calculated for each site: 71.37 kg/m^2^ for site A, 71.02 kg/m^2^ for site B, and 52.29 kg/m^2^ for site C. For waste concrete, WGR was calculated to be 32.62 kg/m^2^ for site A, 36.84 kg/m^2^ for site B, and 27.80 kg/m^2^ for site C. For mixed waste, WGR was calculated to be 31.01 kg/m^2^ for site A, 29.98 kg/m^2^ for site B, and 17.78 kg/m^2^ for site C. The other CW types exhibited significantly low WGRs compared to waste concrete or mixed waste. When the WGRs of each site were compared, sites A and B showed similar patterns in terms of CW WGR by type, but site C exhibited relatively low WGR. This appears to be because the buildings at site C had a higher number of floors than the other sites and the completion of their construction was more recent compared to the other sites. There is also a possibility that the thicknesses of the walls and floors decreased because the same major construction company used more advanced construction technology.

### 4.2. Analysis of the Accumulated Amount of CW Generated during the Construction Period

**Figure 3:**
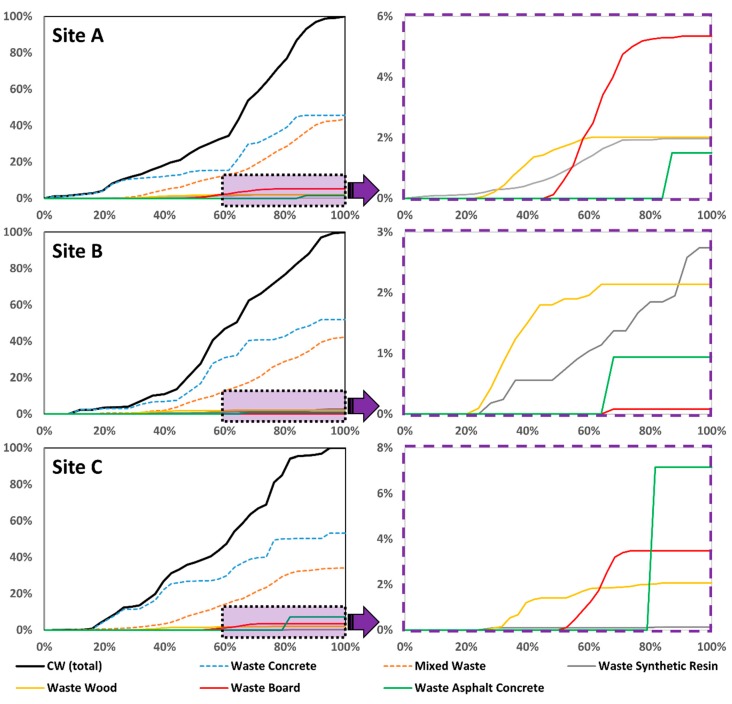
Variation in CW emissions during the construction period.

This section presents an analysis on the six CW types generated at each site. Figure 3 shows the accumulated amount of CW generated at each site during the construction period. The accumulated generation rate of CW exhibited similar S-shaped curves for each site. Waste concrete and mixed concrete were generated throughout the construction period of each site, so the accumulated generation amount increased from beginning to end. The other CW types were generated only in some segments in the middle or at the end of the construction period, which is reflected in the graphs by the sudden increase in the middle.

### 4.3. Analysis of CW Generation Characteristics over the Construction Period

**Figure 4:**
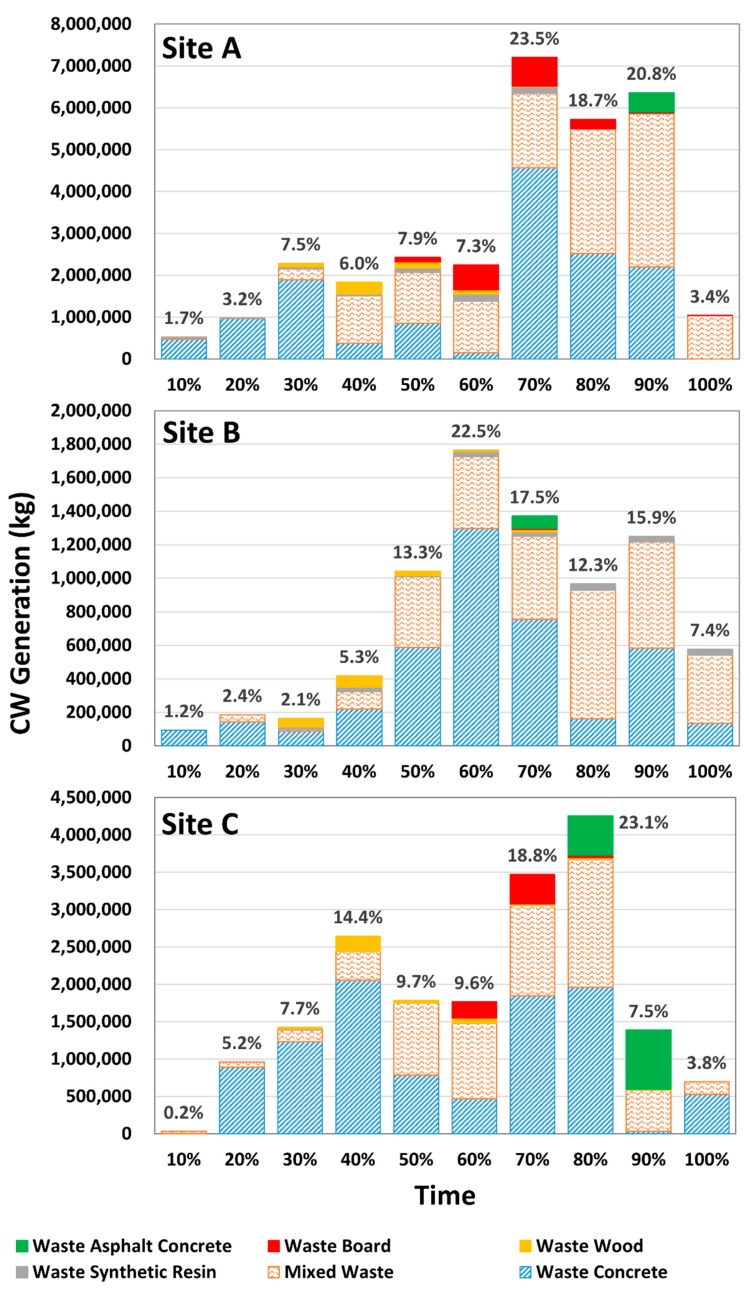
Types and emissions of CW during the construction period of each site.

The total construction period of each site was divided into 10% segments to analyze the CW generation characteristics. Figure 4 shows the amount of CW generated at each site during the construction period. For site A, the largest CW amount (23.5% of the total) was generated in the 60–70% segment, and the smallest CW amount (1.7%) was generated in the 0–10% segment. For site B, the largest CW amount (22.5%) was generated in the 50–60% segment, and the smallest CW amount (1.2%) was generated in the 0–10% segment. For site C, the largest CW amount (23.1%) was generated in the 70–80% segment, and the smallest CW amount (0.2%) was generated in the 0–10% segment. For all three sites, less CW was generated at the beginning and end of the construction period, while more CW was generated in the middle.

For all three sites, the smallest amount of CW was generated in the 0–10% segment, and the amount slowly increased over time. For site A, the amount of CW generated was 3.2% (waste concrete: 3.15%, mixed waste: 0.01%, and waste synthetic resin: 0.04%) in the 10–20% segment, 7.5% (waste concrete: 6.17%, mixed waste: 0.87%, waste synthetic resin: 0.16%, and waste wood: 0.27%) in the 20–30% segment, 6.0% (waste concrete: 1.24%, mixed waste: 3.69%, waste synthetic resin: 0.15%, and waste wood: 0.90%) in the 30–40% segment, 7.9% (waste concrete: 2.78%, mixed waste: 3.96%, waste synthetic resin: 0.36%, waste wood: 0.47%, and waste board: 0.35%) in the 40–50% segment, and 7.3% (waste concrete: 0.50%, mixed waste: 4.00%, waste synthetic resin: 0.56%, waste wood: 0.34%, and waste board: 1.93%) in the 50–60% segment, which indicates a slow increase. For site B, the amount of CW generated was 2.4% (waste concrete: 1.83% and mixed waste: 0.54%) in the 10–20% segment, 2.1% (waste concrete: 1.12%, mixed waste: 0.11%, waste synthetic resin: 0.21%, and waste wood: 0.64%) in the 20–30% segment, 5.3% (waste concrete: 2.80%, mixed waste: 1.33%, waste synthetic resin: 0.34%, and waste wood: 0.87%) in the 30–40% segment, 13.3% (waste concrete: 7.51%, mixed waste: 5.36%, waste synthetic resin: 0.09%, and waste wood: 0.34%) in the 40–50% segment, which also indicates a slow increase. For site C, the amount of CW generated was 5.2% (waste concrete: 4.84% and mixed waste: 0.38%) in the 10–20% segment, 7.7% (waste concrete: 6.68%, mixed waste: 0.84%, waste synthetic resin: 0.10%, and waste wood: 0.11%) in the 20–30% segment, 14.4% (waste concrete: 11.16%, mixed waste: 2.08%, and waste wood: 1.12%) in the 30–40% segment, 9.7% (waste concrete: 4.26%, mixed waste: 5.25%, and waste wood: 0.18%) in the 40–50% segment, and 9.6% (waste concrete: 2.54%, mixed waste: 5.49%, waste wood: 0.39%, and waste board: 1.17%) in the 50–60% segment, which also indicates a slow increase.

For all three sites, the amount of CW generated sharply increased in the middle of the construction period. For site A, the amount of CW generated was 25.5% (waste concrete: 14.92%, mixed waste: 5.81%, waste synthetic resin: 0.52%, waste wood: 0.02%, and waste board: 2.24%) in the 60–70% segment, 18.7% (waste concrete: 8.62%, mixed waste: 9.69%, waste synthetic resin: 0.05%, and waste board: 0.73%) in the 70–80% segment, and 20.8% (waste concrete: 7.19%, mixed waste: 11.94%, waste synthetic resin: 0.04%, waste board: 0.10%, and waste asphalt concrete: 1.50%) in the 80–90% segment. For site B, the amount of CW generated was 22.5% (waste concrete: 16.55%, mixed waste: 5.48%, waste synthetic resin: 0.40%, and waste wood: 0.11%) in the 50–60% segment, 17.5% (waste concrete: 9.63%, mixed waste: 6.34%, waste synthetic resin: 0.33%, waste wood: 0.18%, waste board: 0.08%, and waste asphalt concrete: 0.94%) in the 60–70% segment, 12.3% (waste concrete: 2.07%, mixed waste: 9.79%, and waste synthetic resin: 0.48%) in the 70–80% segment, and 15.9 (waste concrete: 7.44%, mixed waste: 8.09%, and waste synthetic resin: 0.42%) in the 80–90% segment. For site C, the amount of CW generated was 18.8% (waste concrete: 10.02%, mixed waste: 6.59%, waste wood: 0.08%, and waste board: 2.15%) in the 60–70% segment, and 23.1% (waste concrete: 10.65%, mixed waste: 9.27%, waste synthetic resin: 0.01%, waste wood: 0.13%, waste board: 0.16%, and waste asphalt concrete: 2.86%) in the 70–80% segment.

Finally, for all three sites, the amount of CW generated sharply decreased at the end of the construction period. For site A, the amount of CW generated was 3.4% (mixed waste: 3.38% and waste board: 0.01%) in the 90–100% segment. For site B, the amount was 7.4% (waste concrete: 1.72%, mixed waste: 5.18%, and waste synthetic resin: 0.47%) in the 90–100% segment. For site C, the amount was 7.5% (waste concrete: 0.17%, mixed waste: 2.99%, waste synthetic resin: 0.01%, waste wood: 0.06%, and waste asphalt concrete: 4.29%) in the 80–90% segment and 3.8% (waste concrete: 2.85% and mixed waste: 0.93%) in the 90–100% segment.

Waste concrete and mixed waste were generated throughout the construction period for all sites. Waste synthetic resin was generated throughout the construction period at sites A and B but only during specific segments at site C. This appears to be because of the difference in CW treatment methods at each site. More waste wood was generated in the 30–40% segment at all sites. This may be because waste wood was mainly generated from wooden pallets, so it was generated from the reinforced concrete construction and other building finishing works that corresponded to the segment. More waste board was generated in the 60–70% segment of the construction period for all sites, which corresponded to the residue of the gypsum boards used in the finishing works performed during this time. Waste asphalt concrete was generated during different segments at the three sites. This appears to be because waste asphalt concrete was generated by road pavement construction, which is usually performed at the end of the building construction, and building finishing works were performed at different times at each site.

The average WGR of each segment was calculated based on the amount of each CW type generated from the three sites. Table 6 presents the calculated WGR throughout the construction period. The WGR varied widely for each segment with a range of 0.72–13.02 kg/m^2^. In the 0–10% segment, WGR was as low as 0.72 kg/m^2^ and was mostly due to waste concrete (0.65 kg/m^2^). In the 10–40% segments, WGR values were 2.23, 3.62, and 5.19 kg/m^2^, which were less than 10% of the WGR for the total construction period. They were mostly caused by waste concrete (2.03, 2.90, and 2.90 kg/m^2^). In the 40–90% segments, the WGR of each segment (6.72–13.02 kg/m^2^) was greater than 10% of the WGR for the total construction period. In other words, these segments generated relatively more CW than the other segments for the total construction period. In the 90–100% segment, WGR was 3.21 kg/m^2^, which was relatively low compared to the previous segments because the entire construction was being finished. With regard to the CW types, waste concrete (32.42 kg/m^2^), mixed waste (26.26 kg/m^2^), and waste synthetic resin (1.14 kg/m^2^) were generated throughout the construction period, while waste wood (1.35 kg/m^2^), waste board (1.90 kg/m^2^), and waste asphalt concrete (1.83 kg/m^2^) were generated only in specific segments.

### 4.4. Comparison with WGRs of Previous Studies

The calculated WGR results in Table 6 were compared with the data of previous studies presented in Section 2.3. The finding that waste concrete represented the highest proportion of the total CW was very similar to the patterns found in previous studies. However, the results of this study significantly differed in terms of the CW type classification and amount of CW generated. This appears to be because of the following reasons. Most of the previous studies adopted an IM method rather than the DM method, so their results were predicted values based on theoretical DBs rather than the actual measurements of the CW amount generated. Moreover, most of the previous studies proposed WGR a long time ago. Therefore, their results do not properly reflect the changes in CW types and amounts of CW generated caused by the development of demolition technology. Among previous studies, Shim et al. (2014) [39] also calculated the WGR with DM, and their CW type classification and WGR calculation results for each type (except waste soil and stone and construction sludge) were similar to the results of the present study.

## 5. Conclusions

Information on CW generation is very important for its efficient management. Previous studies were based on theoretical DBs and have limited ability to show changes in the CW generation amount during the construction period at a site. In this study, the CW data generated at recently completed apartment building sites were collected through DM to calculate the WGR of each CW type during the construction period. The CW generation characteristics of each type throughout the construction period were analyzed and compared with the results of previous studies.

In this study, CW was classified into six types (waste concrete, waste asphalt concrete, waste wood, waste synthetic resin, waste board, and mixed waste). The WGR was calculated to be 71.37 kg/m^2^ for site A, 71.02 kg/m^2^ for site B, and 52.29 kg/m^2^ for site C. For all sites, waste concrete represented the highest proportion of the amount of CW generated (45.7%, 51.9%, and 53.2%, respectively) followed by mixed waste (43.4%, 42.2%, and 34.0% respectively). The amount of CW generated was lowest at the beginning of the construction period for all three sites. It slowly increased over time and then decreased at the end. Waste concrete and mixed waste were generated throughout the construction period, while the other CW types were generated only in some segments in the middle or at the end. When the WGR values calculated in this study were compared with the results of previous studies, there were significantly large differences in terms of the CW type classification and amount of CW generated, but a recent study that also used DM exhibited similar results to those of this study.

The results of this study have limited representativeness for all buildings because of the difficulty of collecting CW data through DM, hence only three apartment building sites were considered. Further studies on WGR are needed that consider apartment buildings as well as buildings with different sizes and other purposes. Nevertheless, the results of this study are significant when compared to the results of previous studies conducted with IM. In addition, the research method and results of this study are significant in that the construction period was included in the analysis, which could not be considered in previous studies on WGR. Therefore, it is expected that they will be used as basic data for future studies on the optimal resource circulation of CW according to the construction period (e.g., landfill, incineration, recycling, and LCA) in connection with BIM/GIS. The research method and results of this study should contribute to developing efficient CW management systems. Moreover, it is expected that the difference in the generation amount by waste type according to the construction period will significantly contribute to the environment of construction sites and public health (e.g., placement of waste management personnel according to the construction period, management of trucks (schedule and quantity) for the disposal of waste, and the health of workers and nearby residents through linkage with nearby waste treatment facilities).

## Figures and Tables

**Table 1:** Advantages and disadvantages of methods for quantifying construction waste (CW).

Method		Pros and Cons	
**Indirect measurement**	Amount of waste generated is calculated with WGR DBs from the literature and previous studies	Pros	● Various data can be used.
		Cons	● DB selection is difficult because DBs are significantly different. ● It is difficult to reflect differences in the amount of waste generated depending on the structure and construction type
	Amount of waste generated is calculated from the input amounts of materials and addition rate of each material	Pros	● The amount of waste generated can be predicted according to the site characteristics.
		Cons	● Accurate application of material addition rates and weight conversion factors is difficult. ● Amount of waste generated is predicted for a theoretical situation and may differ from the actual amount. ● Various parameters of the actual demolition site cannot be considered.
Direct measurement	Amount of waste generated is directly measured.	Pros	● Data accuracy for the amount of waste generated is very high. ● Actual site situation is properly reflected.
		Cons	● Much time and manpower are required. ● When waste types are mixed during waste generation, it is difficult to identify the amount generated by type.

WGR, waste generation rate; DB, database.

**Table 2:** Comparison of waste generation rates (WGRs) in previous studies (unit: kg/m^2^).

Researchers		a	b	c	d	e	f	g	h	i	j	k	l	m
Year		1995	1995	1997	1999	2004	2007	2007	2011	2012	2013	2013	2014	2014
Country		Korea	Korea	Korea	Korea	Korea	Norway	USA	Spain	Malaysia	China	Spain	Korea	Korea
Combustible	Waste wood	8.647	3.4		7.22	1.69	2.75	6.40	4.80	8.16	7.61		4.16	0.3084
	Waste synthetic resin	5.618	5.6		2.90			0.49	1.80	0.48			0.67	0.3621
	Waste fiber	0.574	0.622		0.32									
	Waste wallpaper	2.638	2.8		0.06		0.46	4.90	0.36					0.0817
Incombustible	Waste concrete	28.71	13	20	15.87	25.96	19.11	22.90	79.20	28.80	17.70	64.00	26.7	28.0109
	Waste asphalt concrete	0.351	0.369		0.35			1.50	6.00		3.42	0.1267		
	Waste brick	0.453	17.4		4.53					1.44	3.42			2.2541
	Waste block													0.211
	Waste roofing tile													
	Waste soil and stone ①								10.80					0.0163
	Construction sludge ②									7.20		4.90		
	Waste metal	9.474	10.3	2	5.17	0.05	0.48	0.90	3.00	0.96	3.99	0.75		0.1509
	Waste glass	0.12	0.18		0.12		0.12		0.60					
	Waste tile	0.333	0.325		0.33					0.48	0.49			0.2739
Mixed	Waste board						1.59	1.30	0.24			0.868	0.39	0.4502
	Waste panel													
	Mixed Waste			8.3	1.43					0.48		0.50	26.5	0.9513
Etc.	Etc.	9.522	0.57		9.52	1.54	6.26	0.93	13.20		4.07	8.607		
Total		66.44	54.566	30.3	47.82	29.24	30.77	43.70 (+10% additions)	120.00	48.00	40.70	79.75	58.42	33.0708
Total (except ① & ②)		66.44	54.566	30.3	47.82	29.24	30.77	43.70	109.20	40.80	40.70	74.85	58.42	33.0545

a: Han et al. [30], b: Jung et al. [31], c: Kim et al. [32], d: Seo and Hwang [33], e: Lee et al. [34], f: Bergsdal et al. [35], g: Cochran et al. [27], h: Llatas [36], i: Lachimpadi et al. [28], j: Li et al. [37], k: Mercader-Moyano and Ramírez-de-Arellano-Agudo [38], l: Shim et al. [39], m: Park. [40]; The ① and ② were excluded from the main analysis.

**Table 3:** Outline of survey and three new apartment construction sites (A, B, and C).

Classification	Site A	Site B	Site C
Building purpose	Apartments	Apartments	Apartments
Completion date	June 2012	September 2012	November 2015
Construction period (months)	31	25	38
Number of buildings	21	8	17
Total area (m^2^)	429,270	110,295	352,414
Scale	2 underground floors 12–28 aboveground floors	1 underground floor 14–25 aboveground floors	2 underground floors 25–34 aboveground floors

**Table 4:** CW type and generation for each site (unit: kg).

Site	Unit	Total CW	Type of Material					
			Waste Concrete	Mixed Waste	Waste Synthetic Resin	Waste Wood	Waste Board	Waste Asphalt Concrete
Site A	(kg)	30,636,880	14,004,600	13,310,890	603,280	618,510	1,638,550	461,050
	(%)	100	45.7	43.4	2.0	2.0	5.3	1.5
Site B	(kg)	7,832,870	4,063,520	3,306,900	214,770	167,500	6,560	73,620
	(%)	100	51.9	42.2	2.7	2.1	0.1	0.9
Site C	(kg)	18,426,930	9,797,620	6,265,430	22,390	381,590	642,050	1,317,850
	(%)	100	53.2	34.0	0.1	2.1	3.5	7.2

**Table 5:** WGR by each site (unit: kg/m^2^).

Type of Material	Site A	Site B	Site C
Waste concrete	32.62	36.84	27.80
Mixed waste	31.01	29.98	17.78
Waste synthetic resin	1.41	1.95	0.06
Waste wood	1.44	1.52	1.08
Waste board	3.82	0.06	1.82
Waste asphalt concrete	1.07	0.67	3.74
Total	71.37	71.02	52.29

**Table 6:** CW generation for each type during the construction period (unit: kg/m^2^).

Time	Waste Concrete	Mixed Waste	Waste Synthetic Resin	Waste Wood	Waste Board	Waste Asphalt Concrete	Total
10%	0.65	0.05	0.02	0.00	0.00	0.00	0.72
20%	2.03	0.20	0.01	0.00	0.00	0.00	2.23
30%	2.90	0.38	0.11	0.24	0.00	0.00	3.62
40%	2.90	1.55	0.12	0.62	0.00	0.00	5.19
50%	3.18	3.12	0.11	0.23	0.08	0.00	6.72
60%	4.48	3.20	0.23	0.17	0.66	0.00	8.75
70%	7.58	4.03	0.20	0.06	0.93	0.22	13.02
80%	4.30	6.24	0.13	0.02	0.20	0.50	11.39
90%	3.50	5.28	0.11	0.01	0.02	1.11	10.03
100%	0.90	2.19	0.11	0.00	0.00	0.00	3.21
Total	32.42	26.26	1.14	1.35	1.90	1.83	64.89

## Supplementary Materials

The following are available online at https://www.mdpi.com/1660-4601/16/18/3485/s1, Table S1: Monthly CW generation of site A, Table S2: Monthly CW generation of site B, Table S3: Monthly CW generation of site C.

## Abbreviations

BIM	Building Information Modeling
C&D	Construction and Demolition
C&DW	Construction and Demolition waste
C&DWM	Construction and Demolition Waste Management
CW	Construction Waste
DB	Database
DM	Direct Measurement
GFA	Gross Floor Area
GIS	Geographic Information System
IM	Indirect Measurement
LCA	Life Cycle Assessment
WGR	Waste Generation Rate
