# Supplementary material for: Analysis of Waste Generation Characteristics during New Apartment Construction—Considering the Construction Phase
Source: Int J Environ Res Public Health. 2019 Sep 19;16(18):3485. doi: 10.3390/ijerph16183485 (PMC6765970; doi:10.3390/ijerph16183485)
Supplement: Supplementary file 1 [file ijerph-16-03485-s001.pdf]

**Table S1.** Monthly CW generation of site A

| Month | Type of material | | | | | |
|---|---|---|---|---|---|---|
| | Waste Concrete | Mixed Waste | Waste Synthetic Resin | Waste Wood | Waste Board | Waste Asphalt Concrete |
| 1 | 313,530 | | 11,500 | | | |
| 2 | 21,250 | | 9170 | | | |
| 3 | 116,670 | 30,540 | 6680 | | | |
| 4 | 193,860 | | | | | |
| 5 | 165,280 | | 5620 | | | |
| 6 | 384,760 | | 4670 | | | |
| 7 | 1,202,390 | 19,410 | 6820 | | | |
| 8 | 608,690 | 28,600 | 16,980 | 21,360 | | |
| 9 | 283,570 | 152,840 | 25,060 | 42,130 | | |
| 10 | 115,540 | 228,440 | 6620 | 68,260 | | |
| 11 | 112,960 | 448,310 | 9120 | 104,160 | | |
| 12 | 113,920 | 374,300 | 17,820 | 87,920 | | |
| 13 | 182,610 | 367,630 | 33,460 | 93,070 | | |
| 14 | 163,800 | 208,320 | 25,850 | 20,400 | | |
| 15 | 470,360 | 531,140 | 39,360 | 51,710 | 38,350 | |
| 16 | 217,870 | 506,190 | 49,960 | 34,910 | 136,390 | |
| 17 | 45,150 | 403,210 | 51,420 | 34,930 | 156,560 | |
| 18 | | 343,210 | 62,470 | 41,440 | 272,670 | |
| 19 | | 377,550 | 53,320 | 18,220 | 157,400 | |
| 20 | 2,001,360 | 345,330 | 66,260 | | 284,520 | |
| 21 | 2,405,320 | 600,890 | 41,180 | | 174,060 | |
| 22 | 234,960 | 973,280 | 45,810 | | 235,170 | |
| 23 | 778,990 | 966,920 | 1400 | | 79,100 | |
| 24 | 890,490 | 1,055,190 | 1020 | | 56,710 | |
| 25 | 974,640 | 818,030 | | | 19,950 | |
| 26 | 1,757,090 | 1,208,060 | 11,710 | | 11,500 | |
| 27 | 249,540 | 1,226,970 | | | | 461,050 |
| 28 | | 1,177,320 | | | 16,170 | |
| 29 | | 567,950 | | | | |
| 30 | | 119,840 | | | | |
| 31 | | 231,420 | | | | |
| Total | 14,004,600 | 13,310,890 | 603,280 | 618,510 | 1,638,550 | 461,050 |

**Table S2.** Monthly CW generation of site B

| Month | Type of material | | | | | |
|---|---|---|---|---|---|---|
| | Waste Concrete | Mixed Waste | Waste Synthetic Resin | Waste Wood | Waste Board | Waste Asphalt Concrete |
| 1 | | | | | | |
| 2 | | | | | | |
| 3 | 187,800 | | | | | |
| 4 | | | | | | |
| 5 | 49,150 | 42,550 | | | | |
| 6 | | | | 7,470 | | |
| 7 | | | 14,360 | 26,300 | | |
| 8 | 176,210 | 17,980 | 4550 | 33,110 | | |
| 9 | 106,650 | 71,100 | 24,610 | 29,990 | | |
| 10 | 24,940 | 24,120 | | 21,340 | | |
| 11 | 43,080 | 140,060 | | 22,770 | | |
| 12 | 361,190 | 201,010 | | | | |
| 13 | 368,270 | 156,770 | 13,620 | 7800 | | |
| 14 | 862,840 | 122,670 | 13,480 | | | |
| 15 | 249,210 | 228,000 | 10,890 | 4700 | | |
| 16 | 99,870 | 165,160 | 7790 | 14,020 | | |
| 17 | 642,160 | 195,530 | 18,130 | | 6560 | 73,620 |
| 18 | 24,550 | 271,280 | | | | |
| 19 | | 389,510 | 23,460 | | | |
| 20 | 149,830 | 241,650 | 13,960 | | | |
| 21 | 297,380 | 170,820 | | | | |
| 22 | 150,860 | 275,060 | 7980 | | | |
| 23 | 269,530 | 375,420 | 49,530 | | | |
| 24 | | 169,590 | 12,410 | | | |
| 25 | | 48,620 | | | | |
| Total | 4,063,520 | 3,306,900 | 214,770 | 167,500 | 6560 | 73,620 |

**Table S3.** Monthly CW generation of site C

| Month | Type of material | | | | | |
|---|---|---|---|---|---|---|
| | Waste Concrete | Mixed Waste | Waste Synthetic Resin | Waste Wood | Waste Board | Waste Asphalt Concrete |
| 1 | | | | | | |
| 2 | | | | | | |
| 3 | | 12,050 | | | | |
| 4 | | 23,710 | | | | |
| 5 | | 11,010 | | | | |
| 6 | 65,630 | 11,580 | | | | |
| 7 | 540,450 | 28,340 | | | | |
| 8 | 477,150 | 24,790 | | | | |
| 9 | 430,750 | 40,460 | | | | |
| 10 | 576,290 | 31,100 | 8280 | | | |
| 11 | 23,530 | 36,740 | 9800 | 15,400 | | |
| 12 | 24,290 | 93,550 | | 12,260 | | |
| 13 | 440,140 | 81,910 | | 70,260 | | |
| 14 | 443,980 | 105,790 | | 25,870 | | |
| 15 | 1,028,890 | 102,810 | | 97,570 | | |
| 16 | 641,990 | 182,940 | | 26,370 | | |
| 17 | 101,240 | 268,300 | | 12,250 | | |
| 18 | 145,380 | 335,810 | | | | |
| 19 | 24,160 | 216,860 | | | | |
| 20 | 25,260 | 249,590 | | | 18,920 | |
| 21 | | 225,750 | | 25,850 | 62,570 | |
| 22 | 176,250 | 330,590 | | 28,470 | 75,320 | |
| 23 | 333,090 | 256,040 | | 21,110 | 73,860 | |
| 24 | 859,810 | 303,040 | | 4630 | 88,620 | |
| 25 | 448,630 | 195,320 | | | 145,340 | |
| 26 | 379,570 | 416,880 | | 3840 | 126,150 | |
| 27 | 153,070 | 413,150 | | 4590 | 36,100 | |
| 28 | 27,820 | 311,190 | | 4560 | 15,170 | |
| 29 | 1,743,330 | 518,380 | | 13,000 | | |
| 30 | 130,660 | 574,120 | | 4010 | | |
| 31 | | 349,720 | 4310 | 2500 | | 1,317,850 |
| 32 | 26,820 | 205,940 | | 9050 | | |
| 33 | | 47,870 | | | | |
| 34 | | 63,540 | | | | |
| 35 | 24,460 | 122,090 | | | | |
| 36 | 504,980 | 46,740 | | | | |
| 37 | | 27,730 | | | | |
| 38 | | | | | | |
| Total | 9,797,620 | 6,265,430 | 22,390 | 381,590 | 642,050 | 1,317,850 |
